# Granulocyte-macrophage colony-stimulating factor negatively regulates early IL-10-mediated responses

**DOI:** 10.4155/fsoa-2017-0133

**Published:** 2018-02-14

**Authors:** Ruud HP Wilbers, Lotte B Westerhof, Debbie R van Raaij, Jaap Bakker, Geert Smant, Arjen Schots

**Affiliations:** 1 Wageningen University & Research, Plant Sciences Department, Laboratory of Nematology, Wageningen, The Netherlands

**Keywords:** dendritic cells, granulocyte-macrophage colony-stimulating factor (GM-CSF), interleukin-10 (IL-10), macrophages, signal transduction

## Abstract

**Aim:**

Treatment of inflammatory disorders relies on the intervention in immune responses thereby restoring homeostasis. IL-10 is a cytokine with therapeutic potential, but until now has not been as successful as previously anticipated. A reason for this may be that IL-10 responsiveness depends on the environment of the inflamed tissue. In this study we investigated whether GM-CSF is able to influence IL-10-mediated responses.

**Methodology:**

Dendritic cells and macrophages were differentiated from mouse bone marrow and treated or depleted from GM-CSF prior to analyze their response to IL-10. Activity was assessed by measuring cytokine expression upon lipopolysaccharide stimulation, IL-10-induced signaling and down-stream gene expression.

**Conclusion:**

This study describes that GM-CSF negatively regulates IL-10-mediated responses.

## Lay abstract

Over the last couple of decades inflammatory disorders, like autoimmune disease and allergies, are becoming more prevalent in the western world. These inflammatory disorders are characterized by uncontrolled immune responses against harmless antigens or commensal bacteria. Treatment of these diseases relies on the intervention in inflammatory responses and thereby restoring the balance of the immune system. One approach used in the clinic to balance the immune system is by treating patients with molecules used by our own immune system to suppress inflammation, such as IL-10. However, treatment with IL-10 has not been as successful as previously anticipated. In this study we show that a particular signaling molecule of the immune system that contributes to inflammation negatively affects the response of immune cells toward IL-10 and thereby could contribute to the low efficacy of IL-10 treatment.

The prevalence of inflammatory disorders, like multiple sclerosis, rheumatoid arthritis, inflammatory bowel disease or allergies, has drastically increased over the last couple of decades. These inflammatory disorders are characterized by uncontrolled immune responses against harmless antigens or commensal bacteria. Treatment of these diseases relies on the intervention in inflammatory responses and thereby restoring immune homeostasis. Many inflammatory disorders are treated with immunosuppressive drugs or monoclonal antibodies that target key proinflammatory cytokines, such as TNF-α. However, immune intervention can also be performed with anti-inflammatory cytokines. One such cytokine capable of restoring homeostasis is IL-10. IL-10 is an anti-inflammatory cytokine that suppresses the activity of both antigen-presenting cells and lymphocytes, and has been considered as a promising therapy for several inflammatory disorders [1]. However, until now IL-10 therapy has not been as successful as previously anticipated.

Systemic administration of recombinant human IL-10 produced in *Escherichia coli* has been studied in Phase II clinical trials to treat patients with Crohn's disease. But, even though IL-10 treatment is well tolerated [2], patients seem to respond differently to IL-10 treatment [3]. Several explanations for why IL-10 treatment has not been a successful therapy for Crohn's disease have been postulated: systemic administration does not result in an effective dose in the intestine; disease phenotype/severity cause differences in response to IL-10; IL-10 treatment is only successful as preventive therapy; treatment with IL-10 alone is not sufficient; or IL-10's immunostimulatory effects counterbalance its immunosuppressive effects [4]. To circumvent systemic administration of IL-10, *Lactococcus lactis* was engineered to secrete human IL-10 and used as an oral delivery system [5]. This local delivery system for IL-10 was shown to be successful in two mouse models of intestinal inflammation [5] and was proven to be safe in a small Phase I human trial [6]. However, in 2009 a Phase II human trial with engineered *L. lactis* in patients with ulcerative colitis was completed, but no significant difference in mucosal healing versus a placebo control was observed (ActoGeniX press release). Future research has to address whether local delivery of IL-10 is indeed a promising therapeutic approach.

Another explanation for IL-10's lack of efficacy as therapy could arise from differences between patients depending on disease phenotype or severity [4]. For instance, alveolar macrophages have the reduced ability to respond to IL-10 during chronic inflammation [7]. Pretreatment of these macrophages with TNF-α reduced their ability to respond to IL-10 without affecting IL-10 receptor (IL-10R) expression. Similarly, agonists of Toll-like receptors (TLR) were able to reduce IL-10-mediated phosphorylation and nuclear translocation of the transcription factor STAT3 in macrophages and dendritic cells without lowering surface IL-10R expression [8,9]. In contrast, IL-10R expression and subsequent IL-10R signaling were reduced in macrophages upon recognition of zymosan [10] or ligation of Fc receptors [11]. Furthermore, cytokines, like IFN-γ, have also been shown to alter IL-10-mediated responses [12]. Altogether, there is quite some evidence that inflammatory mediators can influence IL-10 activity.

Previously we described that bone marrow-derived dendritic cells respond differently to IL-10 when compared with macrophages [13]. Dendritic cells have a reduced capacity to suppress lipopolysaccharide (LPS)-induced TNF-α, especially when evaluating early IL-10-mediated suppression. The lack of early TNF-α suppression coincided with the impaired ability of dendritic cells to upregulate *socs3* expression upon IL-10 treatment. We therefore continued to investigate the underlying mechanism that explains why dendritic cells respond differently to IL-10 than macrophages. In this study we describe that GM-CSF, the cytokine used to differentiate dendritic cells, is a key factor that negatively regulates IL-10-mediated responses. We also show that GM-CSF regulates IL-10 activity without strongly affecting STAT3 activation. In contrast, GM-CSF induces constitutive phosphorylation of GSK-3β, but whether this is the mechanism by which GM-CSF controls IL-10 activity needs further investigation.

## Materials & methods

### Mice

Wild-type C57BL/6 J mice were bred and maintained under specific pathogen-free conditions in the animal facilities at Wageningen University. All experiments were approved by and conducted in accordance with relevant guidelines and regulations of the institutional animal care body at Wageningen University. The experiments of this specific study were approved by the animal experiments committee (DEC) of Wageningen University & Research.

### Bone marrow cultures

Bone marrow was isolated from the femur and tibia of 6–12 week-old C57BL/6 J mice. Bone marrow-derived macrophages (BMMΦs) were differentiated at 37°C/5% CO_2_ in RPMI-1640 medium containing 4 mM L-glutamine, 25 mM HEPES (Life Technologies, Bleiswijk, The Netherlands) and supplemented with 10% fetal calf serum, 50 μM β-mercaptoethanol, 50 U/ml penicillin and 50 μg/ml streptomycin and 20% spent medium from L929 cells (DSMZ, Braunschweig, Germany). Bone marrow cells were seeded at 1 × 10^6^ cells/ml in 6- or 96-well tissue culture plates and cultured for 6 days, while refreshing medium at day 3. After 6 days of culture ≥95% of the cells expressed the macrophage markers F4/80 and CD11b.

Bone marrow-derived dendritic cells (BMDCs) were differentiated for 10 days as described [14] using 10% spent medium from murine GM-CSF-transfected X63 cells [15]. X63-GM-CSF cells were kindly provided by dr. M Lutz (University of Erlangen-Nuremberg) with approval of dr. B Stockinger (MRC National Institute for Medical Research). Briefly, bone marrow cells were plated at 2 × 10^5^ cells/ml in bacteriological petri dishes and incubated at 37°C/5% CO_2_. At days 3, 6 and 8 medium was refreshed, and at day 10 both adherent and nonadherent cells were harvested. At this time typically approximately 90% of the cells expressed the dendritic cell markers CD11c and MHC class II.

### Flow cytometry

BMDCs were stained in FACS buffer (phosphate buffered saline (PBS) containing 0.1% bovine serum albumin (BSA) and 5 mM EDTA) using the following monoclonal antibodies for cell surface markers (all obtained from eBioscience, Vienna, Austria): phycoerythrin (PE-conjugated anti-CD11b, allophycocyanin (APC)-conjugated anti-F4/80, PE-conjugated anti-CD11c, APC-conjugated MHC-II. Cells were first incubated with Fc receptor block (eBioscience) for 10 min to block any nonspecific binding and subsequent staining steps were performed for 20 min at 4°C, followed by washing with FACS buffer. Stained cells were acquired using a Cyan-ADP Analyzer (Beckman Coulter, Woerden, The Netherlands) and analyzed with FlowJo software (Tree Star, Inc.).

### LPS stimulation assays

BMMΦs were differentiated in 96-well plates and BMDCs were seeded in 96-well plates at a density of 5 × 10^4^/well. Cells were pretreated for 15 min with IL-10 and subsequently stimulated with 100 ng/ml LPS by adding LPS-containing medium. After 2 h or overnight stimulation, supernatants were analyzed for TNF-α using the Ready-Set-Go!^®^ ELISA kit (eBioscience, Vienna, Austria) according to the supplier's protocol.

### IL-10-induced signaling

BMDCs were treated for 20 min with IL-10 (0, 1, 10 or 100 ng/ml). Cells were lyzed using 1× Cell Lysis Buffer (Cell Signaling Technology, Leiden, The Netherlands) and total soluble protein content in the lysates was determined by the BCA method (Pierce). Proteins were separated on 12% Bis-Tris gels (Life Technologies) followed by transfer to a PVDF membrane (Life Technologies) by a wet-blotting procedure. Thereafter the membrane was blocked in PBS (containing 0.1% v/v Tween-20 (Sigma-Aldrich, Zwijndrecht, The Netherlands) and 5% w/v non-fat dry milk powder) for 1 h at room temperature, followed by overnight incubation at 4°C with monoclonal antibodies specific for STAT3, phospho-STAT3^Y705^ or phospho-STAT3^S727^ in PBS (containing 0.1% v/v Tween-20 (Sigma-Aldrich) and 1% w/v BSA). All STAT3 antibodies were obtained from Cell Signaling Technology. An HRP-conjugated donkey anti-rabbit IgG (Jackson ImmunoResearch, Suffolk, UK) was used as a secondary antibody. Band intensities were analyzed with Image J Software.

Cell lysates were also used to investigate the activation of 18 well-characterized signaling molecules using the PathScan Intracellular Signaling Array kit (Cell Signaling Technology) according to the manufacturer's protocol. Blots and arrays were visualized in the G:BOX Chemi System (VWR International, Amsterdam, The Netherlands).

### Quantitative PCR

BMMΦs were differentiated in six well plates and 3 × 10^6^ BMDCs were seeded in six well plates and were treated for 2 h with IL-10 (0, 1, 10 or 100 ng/ml). Cells were then washed with PBS, and mRNA was isolated with the Maxwell^®^ 16 Tissue LEV Total RNA Purification Kit and the Maxwell^®^ 16 instrument (both from Promega, Leiden, The Netherlands). Then cDNA was synthesized using the GoScript™ Reverse Transcription System (Promega) according to the supplier's protocol. Samples were analyzed in triplicate for *socs3* and *hprt* (reference gene) expression by quantitative PCR using ABsolute SYBR Green Fluorescein mix (Thermo Fisher Scientific, Etten-leur, The Netherlands). Fold induction of *socs3* expression was determined by the Pfaffl method [16].

## Data analysis

All data shown in the figures indicate the average of at least three biological replicates (n) that were determined by three technical replicates. In the figure legends n is indicated and error bars indicate the standard error. Significant differences between samples were calculated using the Student's t-test and regarded as significant when p < 0.05. Significant differences are indicated in the figures by asterisks (p < 0.05 [*] or p < 0.01 [**]).

## Results

### IL-10-mediated signaling in dendritic cells & macrophages

**Figure 1.**
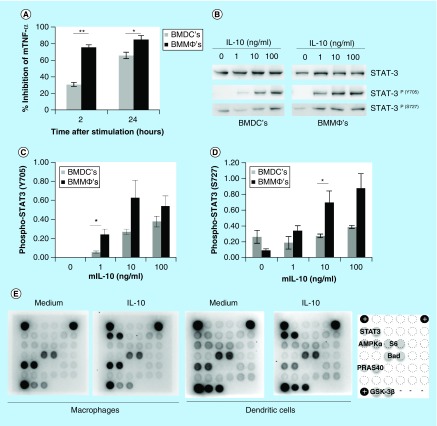
**IL-10-mediated signaling in dendritic cells and macrophages.** **(A)** Macrophages and dendritic cells were pretreated with IL-10 and were stimulated with 100 ng/ml lipopolysaccharide and the inhibition of tumor necrosis factor-α expression was determined at 2 and 24 h (n = 3, error bars represent standard error). **(B)** Phosphorylation of tyrosine 705 (Y705) and serine 727 (S727) of STAT3 by IL-10 (0, 1, 10 and 100 ng/ml) were analyzed in macrophages and dendritic cells using western blot. **(C & D)** Relative induction of STAT3 phosphorylation was quantified for Y705 (C) and S727 (D) upon western-blot analysis (n = 3 or 4 for Y705 and S727, respectively, error bars represent standard error). **(E)** Macrophages and dendritic cells were treated with 100 ng/ml IL-10 and the activation of intracellular signaling pathways was analyzed with a PathScan Array. A representative figure is given for two independent experiments. Asterisk(s) indicate significant differences as determined by a Welch's t-test (*p < 0.05; **p < 0.01).

Previously we observed cell-specific differences in the response to IL-10 in macrophages and dendritic cells [13]. Dendritic cells are strongly impaired in their ability to suppress LPS-induced TNF-α after 2 h of stimulation, whereas macrophages already inhibit TNF-α expression by approxiamtely 75% (Figure 1A). We therefore investigated whether IL-10 signaling differs in these two cell types. First we focused on the activation of the transcription factor STAT3. As shown in Figure 1B, IL-10 induces strong tyrosine phosphorylation of STAT3 (Y705) in a dose-dependent manner in both cell types. Upon quantification of relative STAT3 activation, we observed a higher degree of STAT3^Y705^ phosphorylation in macrophages than in dendritic cells (Figure 1C). However, this was only significantly higher at a dose of 1 ng/ml IL-10. On the other hand, the degree of serine phosphorylation (S727) in macrophages was only significantly higher at a dose of 10 ng/ml (Figure 1D). Yet, when comparing untreated cells with IL-10-treated cells we only observed dose-dependent STAT3^S727^ phosphorylation in macrophages (p < 0.05, for all concentrations). Untreated dendritic cells already have a higher degree of STAT3^S727^ phosphorylation, which is not further enhanced by IL-10 treatment. Serine phosphorylation of STAT3 could therefore be cell-type specific.

Next, we assessed the activation of 18 well-characterized signaling molecules using the PathScan Intracellular Signaling Array kit. In Figure 1E representative arrays are given for macrophages and dendritic cells treated with 100 ng/ml IL-10 and their respective medium controls. The legend indicates which signaling molecules are differentially affected. Strikingly, we only detected phosphorylation of STAT3Y705 upon treatment with IL-10. Interestingly, we did observe differences in the activation of other signaling molecules between untreated macrophages and dendritic cells. Untreated dendritic cells seem to have a higher degree of phosphorylated 5′ adenosine monophosphate-activated protein kinase α (AMPKα) and the ribosomal protein S6, which are indicators for cell-cycle progression and cellular growth. Furthermore, several signaling molecules downstream of the PI3K/Akt pathway (PRAS40, Bad and GSK-3β) are activated in untreated macrophages and dendritic cells, but the degree of GSK-3β phosphorylation (Ser9) is much stronger in dendritic cells. Phosphorylation of GSK-3β inhibits its activity and thereby promotes cell survival. Altogether we conclude that STAT3 is the major downstream signaling molecule for IL-10, but that there are no striking differences in the activation of this transcription factor between macrophages and dendritic cells. However, macrophages and dendritic are differentially affected in signaling molecules that regulate cellular growth and survival.

### GM-CSF negatively regulates IL-10 activity

**Figure 2.**
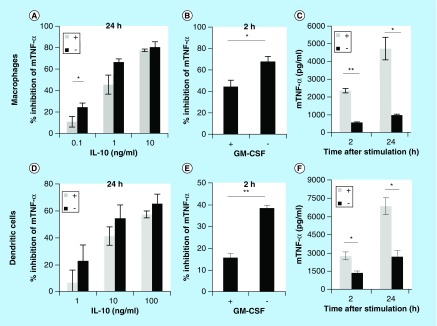
**GM-CSF suppresses early IL-10-mediated responses.** Macrophages **(A–C)** and dendritic cells **(D–F)** were cultured for the last 24 h in the presence or absence (±) of GM-CSF. Cells were pretreated for 20 min with IL-10 followed by stimulation with 100 ng/ml lipopolysaccharide. The inhibition of TNF-α expression was determined after 24 h of stimulation (A & D). Alternatively, cells were pre-treated for 20 min with 10 ng/ml IL-10 and then stimulated with 100 ng/ml lipopolysaccharide. The inhibition of TNF-α expression was determined after 2 h of stimulation (B & E). Absolute expression levels of TNF-α for the abovementioned experiments are also given (C & F). All bars represent the average of 3–5 biological replicates (n = 3–5, error bars indicate standard error) and asterisk(s) indicate significant differences as determined by a Welch's t-test (*p < 0.05; **p < 0.01).

Dendritic cells are differentiated from bone marrow cells by culturing them in the presence of the cytokine GM-CSF. In order to find out if GM-CSF could be responsible for the altered responses of dendritic cells to IL-10, we investigated whether GM-CSF could replicate this phenotype in macrophages. Macrophages and dendritic cells were differentiated from bone marrow, but now GM-CSF was added to macrophages or depleted from dendritic cells for the last 24 h of culture. Cells were then pretreated with IL-10 and subsequently challenged with LPS. Figure 2A–C reveals that GM-CSF is indeed able to alter the response of macrophages toward IL-10. IL-10 inhibits TNF-α expression in a dose-dependent matter in macrophages independent of GM-CSF treatment (Figure 2A), but GM-CSF treatment lowers the maximum percentage of TNF-α inhibition at a lower dose of IL-10. Furthermore, GM-CSF treatment of macrophages significantly reduced early IL-10-mediated suppression of TNF-α by approximately 25% (Figure 2B). We also observed that macrophages treated with GM-CSF produced significantly more TNF-α than untreated macrophages (Figure 2C). These results indicate that GM-CSF is able to alter IL-10-mediated responses in macrophages as was observed for GM-CSF-differentiated dendritic cells.

Similarly, depletion of dendritic cells from GM-CSF enhanced their response toward IL-10. No differences were observed in the suppression of TNF-α expression after 24 h stimulation when dendritic cells were depleted from GM-CSF (Figure 2D). However, early IL-10-mediated suppression of TNF-α expression increased significantly with almost 25% when dendritic cells were depleted from GM-CSF (Figure 2E). Also, production of TNF-α was significantly reduced when dendritic cells were cultured in the absence of GM-CSF (Figure 2F). We, therefore, conclude that GM-CSF is a key factor that modulates early IL-10-mediated responses in both macrophages and dendritic cells.

### GM-CSF regulates IL-10 activity without affecting STAT3 activation

**Figure 3.**
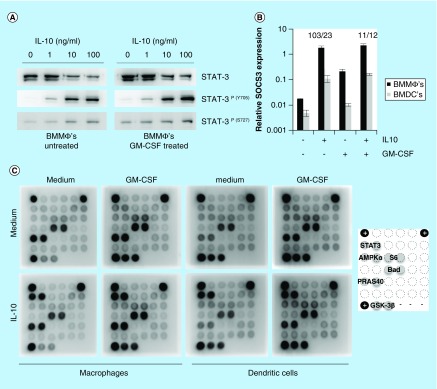
**GM-CSF negatively influences IL-10 activity without affecting STAT3 activation.** **(A)** Macrophages were treated for 24 h with GM-CSF or left untreated. Cells were then activated with IL-10 (0, 1, 10 and 100 ng/ml) and phosphorylation of tyrosine 705 (Y705) and serine 727 (S727) of STAT3 were analyzed by western blot. **(B)** Relative *socs3* transcript levels were analyzed by quantitative PCR in both macrophages and dendritic cells that were treated overnight by GM-CSF or were left untreated (n = 3, error bars represent standard error). Fold induction of *socs3* expression upon IL-10 treatment (100 ng/ml) was calculated using the 2^ΔCt^ method using HPRT as a reference gene and is depicted above the bars in the graph (macrophages/dendritic cells). **(C)** Macrophages and dendritic cells were treated overnight with GM-CSF or left untreated. Cells were then stimulated with 100 ng/ml IL-10 and the activation of intracellular signaling pathways was analyzed by PathScan Array. A representative figure is given for two independent experiments. Asterisk(s) indicate significant differences as determined by a Welch's t-test (*p < 0.05; **p < 0.01).

Early IL-10-mediated responses seem to be controlled by the major anti-inflammatory pathway Jak1-STAT3-SOCS3. To investigate how GM-CSF influences early IL-10-mediated responses we first focused on the activation of STAT3. As shown in Figure 3A, IL-10 induces strong STAT3^Y705^ phosphorylation regardless of GM-CSF treatment. Phosphorylation of STAT3^S727^ seemed also not to be affected by GM-CSF treatment. GM-CSF seems to regulate early IL-10-mediated responses without affecting STAT3 activation.

As STAT3 phosphorylation was not affected by GM-CSF we continued to investigate the effect of GM-CSF on IL-10-induced *socs3* expression. Relative *socs3* transcript levels were determined by quantitative PCR in both macrophages and dendritic cells. As shown in Figure 3B, IL-10 induces *socs3* expression by approximately 100-fold in macrophages, whereas *socs3* upregulation is significantly lower in dendritic cells (12-fold, p = 0.008). Unexpectedly, no significant differences were found for relative *socs3* expression levels upon IL-10 treatment of normal and GM-CSF-treated macrophages. However, the IL-10-specific induction of *socs3* expression was reduced approximately tenfold. This is because GM-CSF itself already enhances *socs3* expression in macrophages prior to IL-10 treatment. Depletion of GM-CSF from dendritic cells only resulted in a twofold increase in *socs3* expression. Therefore, GM-CSF seems to regulate early IL-10-mediated responses not only in a STAT3-independent manner, but also without affecting *socs3* expression levels.

Next, we assessed whether GM-CSF treatment of macrophages alters IL-10-mediated signaling or other signaling pathways by using the PathScan Intracellular Signaling Array kit (Figure 3C). As we observed previously, IL-10 only induces STAT3^Y705^ phosphorylation in macrophages and dendritic cells, which is independent of GM-CSF pretreatment. GM-CSF itself induces an activation state in macrophages that is indistinguishable from dendritic cells. GM-CSF treatment of macrophages enhances the phosphorylation of AMPKα and the ribosomal protein S6, but most strongly induces the phosphorylation of GSK-3β. Phosphorylation of other signaling molecules downstream of PI3K/Akt (PRAS40 and Bad) is also enhanced. On the other hand, depletion of dendritic cells from GM-CSF relieves PRAS40 from phosphorylation, whereas GSK-3β remains strongly phosphorylated. We therefore conclude that GM-CSF alters the activation status of signaling molecules that regulate cellular growth and survival in macrophages and dendritic cells.

## Discussion

IL-10 is an anti-inflammatory cytokine with promising therapeutic potential, but to date IL-10 therapy has not been as successful in the clinic as previously anticipated. A likely explanation for this phenomenon is that IL-10 activity is influenced by the cytokine milieu present in inflamed tissues [10–12]. Previously we have described the differential response of macrophages and dendritic cells toward IL-10 [13]. Dendritic cells are unable to respond rapidly to IL-10 as they are unable to suppress LPS-induced TNF-α at early stages. Furthermore, IL-10-induced *socs3* expression was strongly reduced compared with macrophages. In this study we further investigated the mechanism that controls this differential response between macrophages and dendritic cells. Our study demonstrates that the cytokine GM-CSF, the cytokine used to differentiate dendritic cells, is a key factor that negatively regulates IL-10 activity. GM-CSF pretreatment of bone marrow-derived macrophages also reduced their ability to suppress LPS-induced TNF-α at 2 h after stimulation. Vice versa, depletion of dendritic cells from GM-CSF partially restored their early response to IL-10. Furthermore, both macrophages and dendritic cells cultured in the presence of GM-CSF produced significantly higher levels of TNF-α, which coincided with previous reports from Fleetwood *et al*. [17,18]. Increased TNF-α expression levels upon LPS stimulation are caused by increased basal TNF-α mRNA transcript levels upon GM-CSF treatment [18]. In our study GM-CSF-treated macrophages and dendritic cells do not produce significantly different levels of TNF-α, whereas both cell types respond significantly different toward IL-10. Differences in basal TNF-α expression levels cannot explain the observed differential response toward IL-10. Therefore, we continued to investigate IL-10-mediated signaling in macrophages and dendritic cells.

IL-10 receptor engagement results in the activation of downstream Janus kinases Jak1 and Tyk2, which in turn phosphorylate the transcription factors STAT1, STAT3 and in some cell types STAT5 [19–21]. IL-10 is well known for its ability to activate the Jak-STAT pathway [22], but alternative signaling pathways have been described as well. Especially IL-10-mediated activation of the PI3K signaling pathway has been implicated in IL-10's survival promoting properties [23,24]. Zhou *et al*. also revealed that IL-10 induced the activation of extracellular signal-related kinase [25] one out of two in a PI3K-dependent manner in promyeloid cells [24]. Furthermore, IL-10 has been shown to activate p38 MAPK signaling to induce the expression of heme oxygenase-1 [26]. In our study we could only detect IL-10-mediated activation of the transcription factor STAT3 in bone marrow-derived macrophages and dendritic cells. This indicates that STAT3 is the major transcription factor required for IL-10-mediated responses in bone marrow-derived cells. However, we did observe subtle differences in the phosphorylation of STAT3^Y705^ between regular macrophages and dendritic cells. More striking was the observation that IL-10 was not able to induce STAT3^S727^ phosphorylation in a dose-dependent manner in dendritic cells. Serine phosphorylation of STAT3 has been reported previously for IL-6, IL-22, IFN-y and EGF [27–30] and enhances transcriptional activity of STAT3. The lack of STAT3^S727^ phosphorylation might explain why IL-10-induced *socs3* expression and early inhibition of TNF-α expression are impaired in dendritic cells.

Qasimi *et al*. previously described that SOCS3 is required by IL-10 to suppress TNF-α expression at early time points of stimulation [31]. We therefore investigated the role of SOCS3 in more detail. We observed a reduction in the ability of macrophages to respond rapidly to IL-10 upon GM-CSF treatment, but this seemed to be independent from upregulation of *socs3* expression. Relative induction of *socs3* expression by IL-10 was hardly affected by overnight treatment of macrophages with GM-CSF or depletion of dendritic cells from GM-CSF. This is in contrast to the difference in IL-10-induced *socs3* expression between regular macrophages and dendritic cells. Furthermore, we also did not observe a difference in the phosphorylation of STAT3^Y705^ and STAT3^S727^ between regular macrophages or GM-CSF-treated macrophages. Therefore, GM-CSF seems to negatively regulate early IL-10-mediated responses in macrophages independent from STAT3-induced *socs3* expression.

Interestingly, we did find major differences in the activation status of signaling molecules downstream of PI3K/Akt signaling in macrophages and dendritic cells. Furthermore, GM-CSF induced an activation state in macrophages that was indistinguishable from dendritic cells. Particularly the strong serine phosphorylation of GSK-3β^S9^ was a striking observation in GM-CSF-cultured dendritic cells or GM-CSF-treated macrophages. The degree of GSK-3β^S9^ phosphorylation also seems inversely correlated with early IL-10-mediated responses. A higher degree of GSK-3β^S9^ phosphorylation results in reduced suppression of LPS-induced TNF-α expression by IL-10. As IL-10 is also able to trigger the PI3K signaling pathway it is of great interest to investigate how these signaling pathways of IL-10 and GM-CSF are integrated and result in impaired IL-10-mediated responses.

Activation of STAT3 is regulated by a diverse set of post-translational modifications, including phosphorylation, acetylation and methylation [29,30,32–37]. Every modification plays its own role in regulating optimal STAT3 dimerization, DNA binding activity and transcriptional activity [38]. Involvement of the PI3K signaling pathway in the activation of STAT3 has also been reported previously. Spencer *et al*. revealed that the cytomegalovirus homolog of IL-10 was capable of inducing serine phosphorylation of STAT3^S727^ in a PI3K-dependent manner [39]. Furthermore, acetylation of STAT3^L685^ by IL-6 was also shown to depend on PI3K/Akt activation [40]. However, whether IL-10-induced STAT3 transcriptional activation requires acetylation and/or methylation and whether the PI3K pathway is involved, need further investigation. Interestingly, Waitkus *et al*. recently reported on a novel mechanism of activation of STAT3, which was mediated by GSK-3α/β [38]. GSK-3α/β was shown to directly phosphorylate STAT3 at the residues S727 and T714. The requirement of GSK-3α/β in IL-10-induced STAT3 activation is therefore of great interest as this study shows that GM-CSF strongly inhibits GSK-3β by phosphorylation of serine residue-9. GSK-3α/β might therefore be a key signaling node that is able to control IL-10-mediated responses.

Previously, Hart *et al*. already reported that IL-10 was unable to suppress MHC-II expression in GM-CSF-cultured monocytes [41]. Our study now also demonstrates that GM-CSF is able to alter IL-10-mediated suppression of TNF-α expression in both macrophages and dendritic cells. Furthermore, we and others show that GM-CSF-cultured cells produce significantly higher levels of the proinflammatory cytokine TNF-α [18], but also the secretion of IL-12p70 and IL-23 is significantly enhanced by GM-CSF-treated macrophages [18]. IL-23 has recently been identified as an important proinflammatory cytokine driving both innate and T-cell-induced intestinal inflammation [42]. Therefore, GM-CSF seems to induce a cytokine environment favorable for chronic inflammation. Over the last couple of years GM-CSF has been identified as a key contributor to the development of chronic inflammation in animal models of intestinal inflammation, multiple sclerosis and rheumatoid arthritis [43–45].

## Conclusion

Taking together the key role of GM-CSF in the development of chronic inflammation with the results obtained in this study might explain why IL-10 therapy has not been as effective as previously anticipated. Our study demonstrates that the proinflammatory cytokine GM-CSF is able to negatively regulate IL-10-mediated responses in macrophages and dendritic cells.

## Future perspective

Future research has to elucidate the exact mechanisms by which GM-CSF alters IL-10 signaling pathways and result in impaired cellular responses toward IL-10. It would be interesting to investigate whether GM-CSF alters the phenotype of dendritic cells and macrophages or how GM-CSF signaling interacts with the IL-10 signaling pathway. Ultimately this knowledge could provide us with new therapeutic strategies to treat inflammatory disorders.

**Executive summary**

- IL-10 is a promising immunoregulatory cytokine to be used to treat inflammatory disorders, but its application in the clinic is not as successful as previously anticipated.
- The local inflammatory cytokine environment might affect the response of immune cells toward IL-10.
- Previously we observed that GM-CSF-differentiated dendritic cells from mouse bone marrow respond differently toward IL-10 than bone marrow-derived macrophages.
- Treatment of macrophages with GM-CSF negatively affects their responses toward IL-10.
- Depletion of dendritic cells from GM-CSF restores their response toward IL-10.
- GM-CSF affects early IL-10-mediated responses in particular and does so independent from STAT3 activation and *socs3* expression.
